# New nano-complexes targeting protein 3S7S in breast cancer and protein 4OO6 in liver cancer investigated in cell line

**DOI:** 10.1038/s41598-024-65775-x

**Published:** 2024-07-23

**Authors:** Shaima M. Faheem, Hiam M. Osman, Abdou S. El-Tabl, Moshira M. Abd-El Wahed, Sara M. Younes

**Affiliations:** 1 Department of Chemistry, Faculty of Science, El-Menoufia University, Shebin El-Kom, Egypt; 2 Department of Chemistry, Faculty of Science, Jazan University, Jazan, Saudi Arabia; 3 Department of Pathology, Faculty of Medicine, El-Menoufia University, Shebin El-Kom, Egypt; 4 Chemical Engineering Department, Borg El Arab Higher Institute Engineering and Technology, Alexandria, Egypt

**Keywords:** Schiff-base ether ligand, Complexes, Spectra, Magnetism, Cytotoxic effect, Breast cancer protein 3S7S, Liver cancer protein 4OO6, Molecular docking, Biochemistry, Cancer, Cell biology, Chemical biology

## Abstract

Cancer, a lethal ailment, possesses a multitude of therapeutic alternatives to combat its presence, metal complexes have emerged as significant classes of medicinal compounds, exhibiting considerable biological efficacy, especially as anticancer agents. The utilization of cis-platin in the treatment of various cancer types, including breast cancer, has served as inspiration to devise novel nanostructured metal complexes for breast cancer therapy. Notably, homo- and hetero-octahedral bimetallic complexes of an innovative multifunctional ether ligand (comprising Mn(II), Ni(II), Cu(II), Zn(II), Hg(II), and Ag(I) ions) have been synthesized. To ascertain their structural characteristics, elemental and spectral analyses, encompassing IR, UV–Vis, ^1^H-NMR, mass and electron spin resonance (ESR) spectra, magnetic moments, molar conductance, thermal analysis, and electron microscopy, were employed. The molar conductance of these complexes in DMF demonstrated a non-electrolytic nature. Nanostructured forms of the complexes were identified through electron microscopic data. At ambient temperature, the ESR spectra of the solid complexes exhibited anisotropic and isotropic variants, indicative of covalent bonding. The ligand and several of its metal complexes were subjected to cytotoxicity testing against breast cancer protein 3S7S and liver cancer protein 4OO6, with the Ag(I) complex (7) evincing the most potent effect, followed by the Cu(II) with ligand (complex (2)), Cis-platin, the ligand itself, and the Cu(II)/Zn(II) complex (8). Molecular docking data unveiled the inhibitory order of several complexes.

## Introduction

Metal complexes play a crucial role in the field of medicinal science, the diverse biological functions exhibited by metal ions have significantly contributed to the advancement of therapeutic approaches based on metal compounds. Cisplatin, a prominent medication derived from metals, is extensively utilized in the management of cancer, specifically in the treatment of genitourinary tumors like testicular cancer^1,2^. Metal-based chemotherapeutic agents have been the subject of investigation for potential medical applications. The extensive knowledge possessed by inorganic chemists regarding the coordination and redox properties of metal ions has greatly contributed to the advancement of modern medicinal inorganic chemistry. This field was further stimulated by the fortuitous discovery of cisplatin. Following the identification of suitable ligands for metal ion binding, the characteristics and role of nucleic acids in metal complex formation have been elucidated. Recent reviews have provided a comprehensive overview of the accomplishments in the utilization of metal complexes for medical purposes^3–5^. There has been a growing demand for metal-based compounds in cancer treatment. This surge in demand can be attributed to the prevalence of cancer and, to a lesser extent, the demonstrated in vitro cytotoxic activity of recently synthesized metal-based compounds^6,7^. Schiff base derivatives with a 4-acyl-2-pyrazolin-5-one moiety are a significant class of chemical molecules due to their structural chemistry and biological activity^7,8^. In the realm of anticancer research, pyrazolines have exhibited promising antiproliferative effects against human myelogenous leukemia HL-606. The coordinating ability of the 4-amino-2,3-dimethyl-1-phenyl-3-pyrazolin-5-one ligand has been finely tuned to create a versatile ligand system that can be formed through condensation with a variety of reagents, such as aldehydes, ketones, phenols, carbazides, and others^9–14^. The coordination of metal ions is often intertwined with and influences the biological properties of Schiff bases^15–17^. Cu(II), Ni(II), and Co(II) Schiff base complexes derived from 4,6-diacetylresorcinol have been synthesized and characterized spectroscopically. Furthermore, their cytotoxic activity against MCF-7 cell lines has been investigated^16^. This publication delves into the production of a novel multifunctional ether ligand and its metal complexes as potential anti-breast medicines, as well as the exploration of molecular docking techniques. Molecular docking is a process used to position a ligand molecule on a receptor molecule in order to form a stable compound. This orientation is employed to predict the binding affinity and the strength of the ligand–protein association through a scoring formula. The interaction between the drug and receptor predicts the affinity and activity of the molecule, making it a critical aspect of drug design and discovery. The total free energy of the system is reduced. Discovering and developing new drugs is a challenging endeavor, often aided by the In-Silico technique. Computer-based drug design plays a crucial role in expediting the drug discovery process. It is particularly valuable in the fields of molecular structural biology and computational drug design, allowing for the prediction of the three-dimensional structure of a molecule. Virtual screening is currently performed by ranking candidates docking for large libraries of compounds using the scoring formula^12^.

## Materials and methods

All of the chemicals utilized in the preparation of the ligand and its complexes were of synthetic grade and were employed without any additional purification. The purity of the compounds was confirmed through the utilization of Thin Layer Chromatography (TLC). The analyses of Carbon (C), Hydrogen (H), Nitrogen (N), and Chlorine (Cl) were conducted at the Analytical Unit of Cairo University in Egypt, metal ions were determined using a standard gravimetric technique^10,13^. All metal complexes were subjected to vacuum drying using P_4_O_10_. The measurement of the Infrared (IR) spectra of KBr pellets was performed using a Perkin-Elmer 683 spectrophotometer (4000–400 cm^−1^). The electronic spectra of DMF were obtained using a Perkin-Elmer 550 spectrophotometer. The conductance (10^−3^ M) of the complexes in DMF was measured at 25 °C utilizing a Bibby conductometer type MCl. The ^1^H-NMR spectra of the ligand and its Zn(II) complex were acquired using a Perkin-Elmer R32-90-MHz spectrophotometer with TMS as an internal standard. The mass spectra were captured employing a JEULJMS-AX-500 mass spectrometer equipped with a data system. The thermal studies (DTA and TGA) were conducted in air on a Shimadzu DT-30 thermal analyzer with a heating rate of 10 °C per minute in the range of 27 to 700 °C. The magnetic susceptibilities were measured at 25 °C using the Gouy method and the magnetic susceptibility standard mercuric tetrathiocyanatocobalt(II). The magnetic adjustments were calculated utilizing Pascal's constant. The magnetic moments were computed using a specific equation^10,13^:

$$ \mu_{eff} = 2.84\sqrt {\chi_{M}^{corr} \cdot T} $$

**Figure 1:**
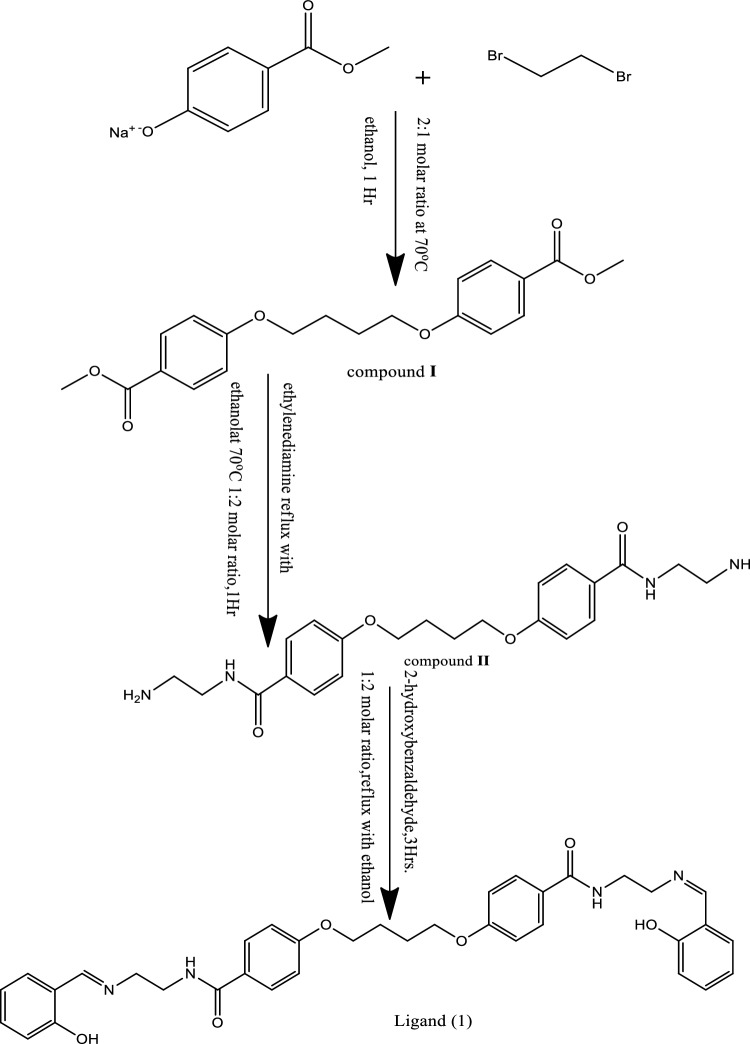
Preparation of ligand [H_4_L] (1).

The Electron Spin Resonance (ESR) spectra of the solid complexes at room temperature were obtained utilizing a Varian E-109 spectrophotometer with 2,2-diphenyl-1-picrylhydrazyl (DPPH) as a reference material. The samples for Transmission Electron Microscopy (TEM) were prepared by depositing colloids onto carbon-coated TEM grids and allowing the liquid carrier to evaporate in air before being examined with a JEOL1230 transmission electron microscope (120 kV). The synthesis of compound (I) and compound (II) [4,4′-(butane-1,4-diylbis(oxy))bis(N-(2-aminoethyl)benzamide)] was performed. Compound I, Fig. 1 was synthesized by adding dibromoethane (1.878 ml, 0.01 mol) to methyl-p-hydroxybenzoate sodium salt (2.49 g, 0.02 mol) dissolved in 50 cm^3^ of pure ethanol and heating with stirring for 1 h. Compound II (product) was obtained by dissolving two moles of ethylenediamine (1.001 g) in 50 cm^3^ of 100% ethanol and subsequently adding it to compound I. The resulting mixture was refluxed on a water bath for an additional hour, then cooled to room temperature, filtered, washed with distilled water, dried, and finally recrystallized from ethanol to yield the pure product.

The Schiff-base ligand (1), known as H_4_L, was synthesized by the combination of two moles of salysaldehyde (1.22 g, 0.01 mol) with one mole of compound II, specifically 4,4′-(butane-1,4-diylbis(oxy))bis(N-(2-aminoethyl)benzamide) (3.30 g, 0.01 mol), in 50 cm^3^ of pure ethanol. Subsequently, the resulting mixture was subjected to reflux with stirring for a duration of 3 h. The black product obtained was then filtered, washed with water, and air-dried to produce a brown product. This product was further subjected to recrystallization from ethanol, resulting in the formation of pristine needle-shaped crystals of (H_4_L)(1). The synthesis of metal complexes (2)–(11) involved the addition of a filtered ethanoic solution (50 cm^3^) of Cu (OAc)_2_·H_2_O (0.6 g, 0.006 mol) to an ethanolic solution (50 cm^3^) of the ligand (1) (1.8 g, 0.006 mol) [1L:2M]. Similarly, complex (2) was formed by combining a solution of Ni (OAc)_2_·4H_2_O (1.28 g, 0.006 mol) with the ligand in a 1L:2M ratio. Complex (3) was synthesized by adding a solution of Hg(OAc)_2_·2H_2_O (3.41 g, 0.006 mol) to the ligand in a 1L:2M ratio. Likewise, complex (4) was obtained through the combination of a solution of Zn(OAc)_2_·4H_2_O (6.15 g, 0.006 mol) with the ligand in a 1L:2M ratio. Complexes (5)–(7) were formed by adding solutions of Mn(OAc)_2_·4H_2_O (6.84 g, 0.006 mol), Ag(OAc). H_2_O (2.70 g, 0.006 mol), and Zn(OAc)_2_·4H_2_O (3 g, 0.006 mol) respectively to the ligand in a 1L:2M ratio. Finally, complex (11) was synthesized by combining solutions of Zn(OAc)_2_·4H_2_O (2.4 g, 0.006 mol) and Cu(OAc)_2_ in a 1L:2M ratio. The resulting mixtures were then refluxed with stirring for a duration of 1–3 h, depending on the type of metal salts used. Subsequently, the colored complexes that formed were filtered, washed with ethanol, and dried under vacuum over P_4_O_10_.

## Biological activity

The evaluation of cytotoxic activity was conducted in the Pathology Laboratory of the Pathology Department at the Faculty of Medicine, El-Menoufia University, Egypt. The ligand and its metal complexes were tested against breast and liver protein cell lines using the Sulfo-Rhodamine-B-stain (SRB) test technique^17,18^. To ensure adhesion to the plate wall, the cells were plated on a 96-multiwell plate (104 cells/well) for 24 h prior to treatment with the compounds. The compounds were administered to the cell monolayer in DMSO (0, 5, 12.5, 25, and 50 g/ml), with triplicate wells created for each dose. The monolayer cells were then incubated with the complexes for 48 h at 37 °C with 5% CO_2_. Following incubation, the cells were fixed, washed, and stained with Sulfo-Rhodamine-B dye. Acetic acid was used to remove excess stain, while Tri EDTA buffer was used to restore attached stain. The intensity of the colors was measured using an ELISA reader. By plotting the relationship between surviving fraction and drug concentration, the survival curve for each tumor cell line after the addition of the specified substance was obtained. Furthermore, molecular docking was performed using specific complexes with breast cancer protein 3S7S and liver cancer protein 4OO6^13^. We select liver cell one time and breast cell one time to have more variation defects in of proteins and cells.

## Results and discussion

**Figure 2:**
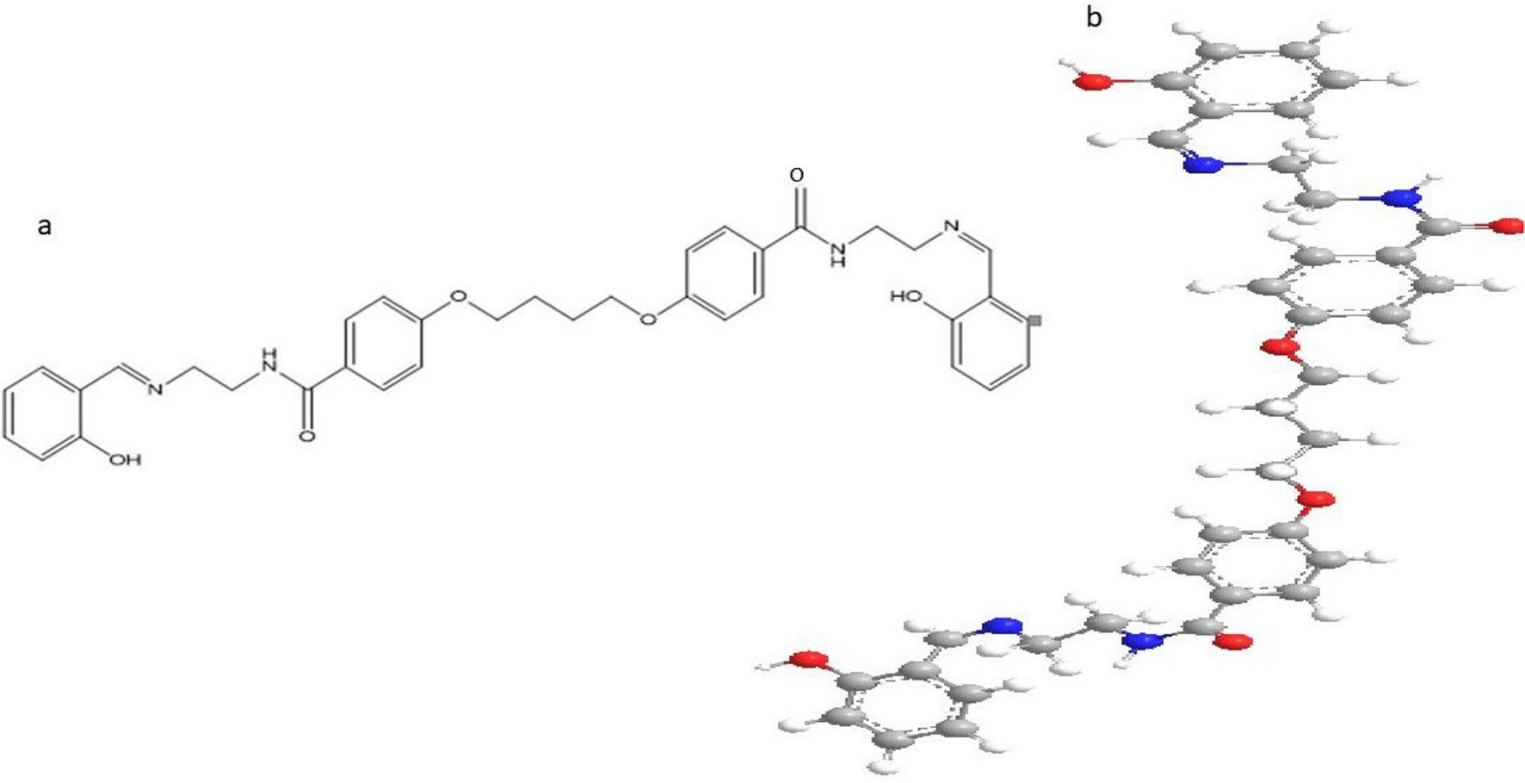
(**a**) Structure of Ligand (I) and (**b**) 3D structure of the ligand (I).

**Figure 3:**
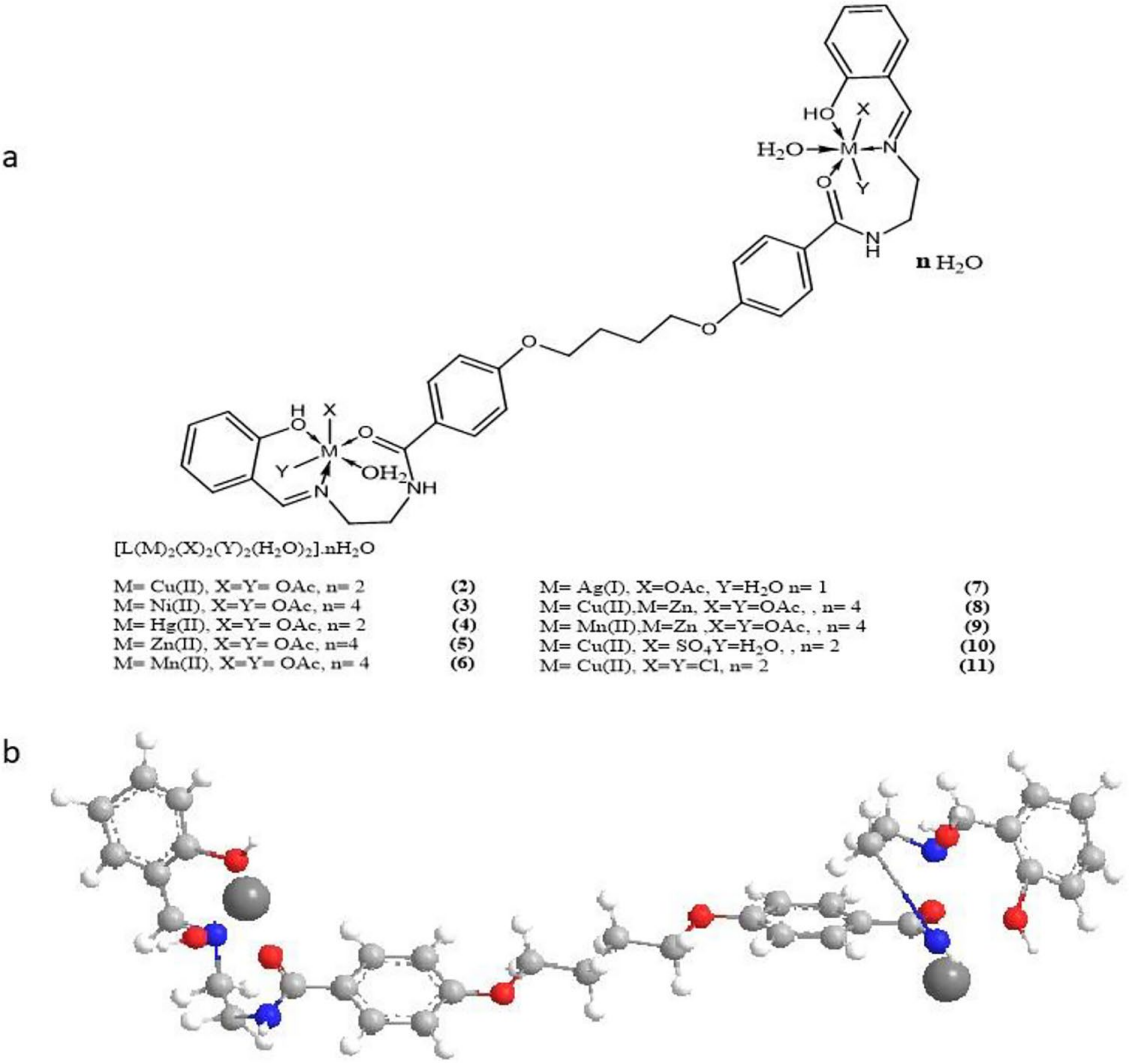
(**a**) Structure representation of Ni(II), Cu(II), Zn(II), Mn(II), (Hg), and Ag(I) complexes and (**b**) 3D metal complexes (general form).

All of the complexes are nanoparticles that are stable at room temperature, non-hydroscopic, insoluble in water, and slightly soluble in common organic solvents like CHCl_3_, but soluble in DMF and DMSO. The analytical and physical data of the ligand and its complexes were provided in Table 1, and the spectral data (Tables 2, 3, 4, 5, 6) were compatible with the proposed structures, as shown in Figs. 2 and 3. Table 1 shows that the molar conductance in DMF solution were in the 6.3–16.4 Ω^−1^cm^2^mol^−1^ range, indicating a non-electrolytic nature^19^, some complexes high values showed partial dissociation in DMF. Complexes (2)–(11) were formed by reacting (1) with metal salts in ethanol at (1L: 2M) molar ratios. The complexes were composed of diatomic complexes.

**Table 1:** Analytical and physical data of [H_4_L], (1) and it's metal complexes: Λ*Ohm^−1^ cm^2^ mol^−1^.

No.	Ligand 1 and complexes	Color	FW	M.P (°C)	Yield (%)	Anal./found (calc.) (%)					Molar conductance Λ*
						C	H	N	M	Cl	
(1)	[(H_4_L) C_36_H_38_N_4_O_6_	Brown	622.28	> 300	88	69.05(69.44)	6.11(6.15)	8.68 (9.00)	–	–	8.2
(2)	[(H_4_L)(Cu)_2_(OAc)_4_(2H_2_O)]. H_2_O C_44_H_58_Cu_2_N_4_O_18_	Dark green	1058.40	> 300	78	49.91(49.95)	5.41(5.53)	5.24 (5.30)	11.94 (12.01)	–	7.56
(3)	[(H_4_L) (Ni)_2_(OAc)_4_(2H_2_O)]0.2H_2_O C_44_H_58_N_4_Ni_2_O_18_	Orange	1038.33	> 300	75	49.99 (50.41)	5.22 (5.58)	4.77 (5.34)	10.25 (11.20)	–	6.38
(4)	[(H_4_L)(Hg)_2_(OAc)_4_(2H_2_O)]0.2H_2_O C_44_H_58_Hg_2_N_4_O_18_	Yellow	1332.13	> 300	77	38.21 (39.67)	4.32 (4.39)	3.98 (4.21)	29.74 (30.12)	–	8.2
(5)	[(H_4_L) (Zn)_2_(OAc)_4_(2H_2_O)]0.2H_2_O C_44_H_58_N_4_O_18_Zn_2_	Faint yellow	1061.71	> 300	75	49.66 (49.78)	4.98 (5.51)	5.22 (5.28)	12.20 (12.32)	–	9.0
(6)	[(H_4_L) (Mn)_2_(OAc)_4_(2H_2_O)]0.3H_2_O C_44_H_60_Mn_2_N_4_O_19_	Faint brown	1058.84	> 300	77	49.12(49.91)	5.63(5.71)	5.25 (5.29)	10.19 (10.38)	–	8.7
(7)	[(H_4_L) (Ag)_2_(OAc)_2_(2H_2_O)].H_2_O C_40_H_50_Ag_2_N_4_O_13_	Golden	1010.58	> 300	82	41.49 (41.53)	4.86 (4.99)	5.51 (5.54)	21.11 (21.35)	–	7.63
(8)	[(H_4_L)(Cu)(Zn)(OAc)_4_(2H_2_O)]0.2H_2_O C_44_H_58_N_4_O_18_CuZn	Dark green	1059.87	> 300	80	49.28 (49.68)	5.48 (5.52)	5.23 (5.29)	11.83 (12.17)	–	7.8
(9)	[(H_4_L)(Mn)(Zn)(OAc)_4_(2H_2_O)]0.2H_2_O C_44_H_58_N_4_O_18_MnZn	Dark oily	1051.27	> 300	70	49.47 (50.27)	5.50(5.56 )	5.29 (5.33)	11.25 (11.45)	–	8.2
(10)	[(H_4_L)(Cu)_2_(SO_4_)_2_(H_2_O)_4_]0.2H_2_O C_36_H_50_Cu_2_N_4_O_20_S_2_	faint oily	1050.20	> 300	78	(41.18) 36.30	3.64 (4.80)	5.66 (5.34)	12.41 (12.10)	–	8.0
(11)	[(H_4_L)(Cu)_2_(Cl)_4_(H_2_O)_2_]0.2H_2_O C_36_H_46_Cl_4_Cu_2_N_4_O_10_	Black	963.68	> 300	75	(44.87) 44.11	4.76 (4.81)	5.68 (5.81)	12.96 (13.19)	14.32 (14.72)	8.6

**Table 2:** IR frequencies of the bands (cm^−1^) of ligand [H_4_L_2_] and its metal complexes and their assignments.

	υ(H_2_O)	υ(H-bond.)	υ(NH)	υ(OH)	υ(C=O)	υ(C=N)	υ(Ar)	υ(OAc)/SO_4_	υ(M–O)	υ(M–N)	υ(M–Cl)
(1)	–	3560–3350 3340–2980	3152	3360, 3350 1185, 1335	1683, 1632	1620, 1605	1459–1460 853, 815	–	–	–	–
(2)	3560–3370 3360–3100	3540–2250 3240–2820	3140	3355–3415 1281, 1330	1685, 1632	1611, 1603	1519, 1440 850, 816	1440, 1320	676	572	–
(3)	3500–3360 3350–3060	3540–3260 3250–2750	3130	3355–3470 1278, 1312	1697, 1644	1617, 1600	1530, 1443 771, 744	1432, 1346	622	542	–
(4)	3490–3370 3360–3050	3540–3270 3260–2790	3138	3450, 3348 1310–1285	1692, 1630	1611, 1602	1585, 1516 768, 746	1496, 1417	661	519	–
(5)	3470–3380 3370–3080	3520–3240 3230–2870	3130	3435, 3339 1172, 1310	1717, 1639	1615, 1600	1544, 1490 768, 751	1445–1336	610	546	–
(6)	3500–3330 3320–3080	3530–3270 3260–2820	3128	3394, 3420 1270, 1311	1710, 1650	1613, 1605	1570, 1506 795, 757	1441, 1338	624	563	–
(7)	3460–3210 3265–3080	3530–3270 3265–2920	3130	3485, 3430 1281, 1332	1797, 1631	1619, 1605	1570, 1496 795, 747	1455, 1360	650	520	–
(8)	3460–3320 3310–3080	3560–3230 3220–2870	3135	3470, 3426 1186, 1333	1717, 1631	1615, 1601	1516, 1418 771, 739	1422, 1402 1320–1340	642	528	–
(9)	3450–3310 3300–3105	3530–3280 3270–2930	3140	3450, 3423 1287, 1334	1683, 1630	1615, 1603	1541, 1 765, 705	1435, 1400 1330, 1320	623	560	–
(10)	3450–3360 3500–3100	3510–2280 3270–2850	3127	3416, 3350 1290, 1335	1718, 1634	1610, 1602	1504, 1470 757, 745	1444, 1146 1087, 742	617	510	–
(11)	3475–3310 3265–3080	3520–3285 3280–2970	3133	3485, 3425 1333, 1393	1684, 1600	1612, 1600	1509, 1442 757, 751	–	614	523	462, 405

**Table 3:** (i) Mass spectrum of the ligand [H_4_L] (1), (ii) Mass spectrum of [(H_4_L)(Ni)_2_(OAc)_4_]·2H_2_O complex (3), (iii) Mass spectrum of [(H_4_L_2_) (Mn)_2_(OAc)_4_]·2H_2_O complex (6).

m/z	Rel. Int	Assignments
(i)		
65	35	N_2_H_5_O_2_
93	25	C_2_H_9_N_2_O_2_
121	100	C_3_H_9_N_2_O_3_
152	19	C_4_H_12_N_2_O_4_
196	15	C_7_H_20_N_2_O_4_
268	24	C_11_H_28_N_2_O_5_
329	22	C_15_H_28_N_3_O_5_
415	17	C_21_H_28_N_4_O_5_
532	35	C_29_H_33_N_4_O_6_
622	19	C_36_H_38_N_4_O_6_
(ii)		
58	34	C_3_NO_2_
121	81	C_4_H_11_NO_3_
324	100	C_15_H_18_NO_7_
500	17	C_24_H_24_N_2_O_10_
581	15	C_29_H_29_N_2_O_11_
669	29	C_35_H_29_N_2_O_12_
726	21	C_38_H_29_N_3_O_12_
802	34	C_40_H_30_N_3_O_15_
962	25	C_42_H_37_N_4_O_17_Ni
1048	38	C_44_H_58_N_4_O_18_Ni_2_
(iii)		
65	30	C_3_HN_2_
121	100	C_5_H_3_N_2_O_2_
179	23	C_7_HN_2_O_4_
330	14	C_14_H_4_NO_8_
445	15	C_20_H_15_NO_10_
583	18	C_24_H_18_NO_12_Mn
649	10	C_27_H_20_N_3_O_14_Mn
795	12	C_33_H_30_N_3_O_18_Mn
960	50	C_37_H_46_N_4_O_19_Mn_2_

**Table 4:** Thermal data for some metal complexes.

Compound no. Molecular formula	Temp. (°C)	DTA (peak)		TGA (Wt. loss %)		Assignments
		Endo	Exo	Calc	Found	
Complex (2) [(H_4_L)(Cu)_2_(OAc)_4_(2H_2_O)]·H_2_O C_44_H_58_Cu_2_N_4_O_18_	55	Endo	–	–	–	Broken of H-bonding
	135	Endo	–	1.70	1.68	Loss of (H_2_O) hydrated water molecules
	260	Endo	–	3.46	3.58	Loss of (2H_2_O) coordinated water molecules
	375	Endo	–	23.50	23.42	Loss of four coordinated acetate group
	540	Endo	–	–	–	Melting point
	415, 490, 680, 840	–	Exo	20.70	20.65	Decomposition process with the formation of 2CuO
Complex (6) [(H_4_L) (Mn)_2_(OAc)_4_(2H_2_O)]·4H_2_O C_44_H_60_Mn_2_N_4_O_19_	50	Endo	–	–	–	Broken of H-bonding
	120	Endo	–	6.09	5.98	Loss of (4H_2_O) hydrated water molecules
	210	Endo	–	2.05	1.98	Loss of (2H_2_O) coordinated water molecules
	370	Endo	–	24.38	24.29	Loss of four coordinated acetate group
	580	Endo	–	–	–	Melting point
	410, 465, 650, 830	–	Exo	23.33	22.88	Decomposition process with the formation of 2MnO
Complex (9) [(H_4_L)(Mn)(Zn)(OAc)_4_(2H_2_O)]·4H_2_O C_44_H_58_N_4_O_18_MnZn	45	Endo	–	–	–	Broken of H-bonding
	85	Endo	–	4.42	4.36	Loss of (4H_2_O) hydrated water molecules
	140	Endo	–	3.55	3.50	Loss of (H_2_O) coordinated water molecules
	280	Endo	–	24.11	24.02	Loss of four coordinated acetate group
	380	Endo	–	–	–	Melting point
	415, 550, 680, 745	–	Exo	20.47	20.38	Decomposition process with the formation of ZnO/MnO
Complex (11) [(H_4_L)(Cu)_2_(Cl)_4_(H_2_O)_2_]·2H_2_O C_36_H_46_Cl_4_Cu_2_N_4_O_10_	48	Endo	–	–	–	Broken of H-bonding
	85	Endo	–	3.47	3.38	Loss of (2H_2_O) hydrated water molecules
	190	Endo	–	3.88	3.81	Loss of (2H_2_O) coordinated water molecules
	315	Endo	–	15.92	15.85	Loss of four coordinated chloride ions
	380	Endo	–	–	–	Melting point
	439, 460, 580	–	Exo	21.21	21.15	Decomposition process with the formation of 2CuO

**Table 5:** The electronic absorption spectral bands (nm) and magnetic moments (B.M.) for the ligand [H_4_L] (1), and its complexes.

No.	λ max (nm)	μeff in B.M	ν2/ν1
(1)	280 nm (log ε = 3.8 × 10^–3^) mol^−1^ cm^−1^, 315 nm (log ε = 4.25 × 10^–3^) mol^−1^ cm^−1^	–	–
(2)	267, 297, 320, 395, 480, 582, 603	1.69	–
(3)	268, 285, 310, 475, 590, 617, 776	3.12	1.24
(4)	270, 290, 312, 460, 570, 615	Diamagnetic	–
(5)	268, 283, 310	Diamagnetic	–
(6)	265, 280, 307, 462, 568, 612	5.61	–
(7)	267, 285, 310	Diamagnetic	–
(8)	265, 299, 392, 415, 533, 610	1.71	–
(9)	260, 283, 318, 420, 560, 605	4.92	–
(10)	265, 280, 305, 478, 575, 602	1.70	–
(11)	266, 282, 307, 462, 578, 605	1.70	–

**Table 6:** ESR data for the metal complexes:

Complex	g||	g_┴_	g_iso_^a^	A_||_ _(G)_	A_┴_ _(G)_	A_iso_^b^ _(G)_	G^c^	ΔE_xy_ _(cm_^–1^_)_	ΔE_xz_ _(cm_^−1^_)_	K_┴_^2^	K||^2^	K^2^	K	g||/A_||_ _(cm_^−1^_)_	α^2^	β^2^	β_1_^2^	− 2β	a^2^d (%)
(2)	2.15	2.05	2.08	95	5	35	3	17,182	20,833	0.6	0.83	0.52	0.72	238.8	0.46	1.3	0.82	175.7	74.76
(6)	–	–	2.02	–	–	–	–	–	–	–	–	–	–	–	–	–	–	–	–
(8)	–	–	2.13	–	–	–	–	–	–	–	–	–	–	–	–	–	–	–	–
(9)	–	–	2.03	–	–	–	–	–	–	–	–	–	–	–	–	–	–	–	–
(10)	2.12	2.04	2.07	100	10	40	3.0	17,391	20,920	0.48	0.31	0.42	0.65	216.3	0.45	1.07	0.69	177.2	75.41
(11)	2.17	2.06	2.09	50	5	20	2.83	–	–	–	–	–	–	431.25	–	–	–	–	–

g_iso_ = (2g_⊥_ + g_||_)/3.

The comparison of the infrared (IR) spectra of the ligand (1) and its metal complexes (2)–(11) provides insight into the bonding mechanism between the ligand and the metal ion. The presence of two types of hydrogen bonding, both intra- and intermolecular, is indicated by bands observed in the 3560–3350 cm^−1^ and 3340–2980 cm^−1^ ranges of the ligand^20^. The higher frequency range corresponds to a weaker hydrogen bond, whereas the lower frequency band is associated with a stronger hydrogen bond. Additionally, a medium band at 3140 cm^−1^ is attributed to the v(NH) group^21,22^. Interestingly, the v(NH) group in the complexes appears at the same location as in the free ligand, indicating that the NH group does not participate in metal ion coordination^23^. The v(NH) band is discovered in the metal complexes within the 3013–2546 cm^−1^ range. Furthermore, the v(OH) group is responsible for a strong band observed at 3360 cm^−1^, which appears in the complexes within the 3480–3420 cm^−1^ range. The v(C=O) group causes a strong band at 1690 cm^−1^, observed in the complexes within the 1685–1644 cm^−1^ range. Bands observed within the ranges of 3560–3210 cm^−1^ and 3370–3820 cm^−1^ in the complexes are attributed to hydrated, coordinated water molecules and hydrogen bonding. Another strong band at 1625 cm^−1^ is caused by the v(C=N) band, which occurs throughout the complex between 1601 and 1620 cm^−1^. The acetate ion band v(Ac) is observed within the range of 1449–1260 cm^−1^, while the sulphate ion band v(Ac) emerges at 1160, 1164, 1040, and 690 cm^−1^. Aromatic bands are observed in two regions: the first within the range of 1570–1460 cm^−1^ and the second within the range of 853–739 cm^−1^ for the ligand. In the complexes, aromatic bands are observed in two regions as well: the first within the range of 1519–1402 cm^−1^ and the second within the range of 771–617 cm^−1^. Specifically, the band at 405 cm^−1^ is attributed to v(Cu–Cl) complex (11)^24,25^. Complexes with bands within the range of 676–610 cm^−1^ are assigned to v(M–O), while bands within the range of 512–510 cm^−1^ are assigned to v(M–N)^26,27^. The ^1^H nuclear magnetic resonance (NMR) spectra of the ligand (1) and its Zn(II) complex (5) reveal significant information. In the ligand's ^1^H-NMR spectra, a peak at 7.7 ppm is observed due to an NH group proton, while peaks within the range of 3.5–4.37 ppm are attributed to methylene group protons. Protons from the OH aromatic group are detected at 10.8 ppm. Due to the presence of strong hydrogen bonding, the protons of aromatic rings are observed at 6.6–7.3 ppm, and the protons of the methine group appear at 8.8 ppm^28,29^. In the ^1^H-NMR spectrum of the complexes (5), NH protons are observed at 7.8 ppm, and methylene protons are observed within the range of 3.56–4.47 ppm. Peaks at 10.5 ppm are due to aromatic OH, while aromatic ring protons emerge within the range of 6.4–7.15 ppm. Finally, peaks at 8.3 and 9.0 ppm are observed due to methane group protons^30,31^. When comparing the ^1^H-NMR of the ligand and the spectra of complex (5), a significant downfield shift of the proton signal in relation to the free ligand is observed, indicating that the metal ions are coordinated to the ligand. This shift could be attributed to the formation of a coordination bond (NM)^32–34^. The proposed formulations of the ligand (1), its Ni(II) complexes (3), and Mn(II) complex (6) are supported by the mass spectra. The spectrum of the ligand shows molecular ion peaks (m/z) at 622 amu, which is consistent with the molecular weight of the ligand (622). Furthermore, the observed fragments at m/z = 65, 93, 121, 152, 196, 268, 329, 415, 532, and 622 amu correspond to N_2_H_5_O_2_, C_2_H_9_N_2_O_2_, C_3_H_9_N_2_O_3_, C_4_H_12_N_2_O_4_, C_7_H_20_N_2_O_4_, C_11_H_28_N_2_O_5_, C_15_H_28_N_3_O_5_, C_21_H_28_N_4_O_5_, C_29_H_33_N_4_O_6_, and C_36_H_38_N_4_O_6_ moieties, respectively. However, the Ni(II) complex (3) exhibits a peak (m/z) at 1048 amu. Additionally, the observed peaks at m/z 58, 121, 324, 500, 581, 669, 726, 802, 926, and 1084 amu are attributed to C_3_NO_2_, C_4_H_11_NO_3_, C_15_H_18_NO_7_, C_24_H_24_N_2_O_10_, C_29_H_29_N_2_O_11_, C_35_H_29_N_2_O_12_, C_38_H_29_N_3_O_12_, C_40_H_30_N_3_O_15_, C_42_H_37_N_4_O_17_Ni, and C_44_H_58_N_4_O_18_Ni_2_ moieties, respectively. Finally, the Mn(II) complex (6) exhibits a peak (m/z) at 960 amu. The observed fragments (m/z) at 65, 121, 179, 330, 445, 583, 649, 795, and 960 amu are due to Assignments C_3_HN_2_, C_5_H_3_N_2_O_2_, C_7_HN_2_O_4_, C_14_H_4_NO_8_, C_20_H_15_NO_10_, C_24_H_18_NO_12_Mn, C_27_H_20_N_3_O_14_Mn, C_33_H_30_N_3_O_18_Mn, and C_37_H_44_N_4_O_18_Mn_2_, respectively. The data is presented in Table 3(i, ii, and iii).

Thermal analyses including differential thermal analysis (DTA) and thermogravimetric analysis (TGA) were carried out in order to validate the presence of water molecules, as indicated by the IR spectra. The thermal stability of complexes (2), (6), (9), and (11) was confirmed within the temperature range of 27–700 °C, with no significant changes observed up to 45 °C. The disruption of hydrogen bonds was observed as an endothermic peak at temperatures ranging from 45 to 55 °C, as shown in Table 4. Subsequently, endothermic peaks were detected within the temperature range of 80–120 °C, corresponding to the loss of hydrated water molecules. Furthermore, the removal of coordinated water molecules occurred in the 140–260 °C range^35–37^. The thermogram of the Cu(II) complex (2) exhibited a disintegration process that took place in six phases. The first phase occurred at 55 °C, resulting in a minor weight loss accompanied by an endothermic peak, possibly attributed to the breaking of hydrogen bonds. The second phase occurred at 135 °C with a weight loss of 1.68% (Calculated: 1.70%) and an endothermic peak, which could be associated with the removal of two hydrated water molecules. The elimination of coordinated water molecules took place during the breakdown process at 260 °C, causing a weight loss of 3.85% (Calculated: 3.46%). The removal of four acetate groups generated an endothermic peak at 375 °C with a weight loss of 23.42% (Calculated: 23.50%). The complex displayed an endothermic peak at 540 °C due to its melting point. Finally, exothermic peaks were observed at 415, 490, 680, and 840 °C, indicating a gradual oxidative thermal breakdown, resulting in a weight loss of 20.65% (Calculated: 20.70%), leaving behind 2CuO^30^. Similarly, the thermogram of the Mn(II) complex (6) revealed a disintegration process occurring in six stages. The first stage took place at 50 °C, resulting in a minor weight loss accompanied by an endothermic peak, potentially associated with the disruption of hydrogen bonds. The second stage occurred at 120 °C, leading to a weight loss of 4.98% (Calculated: 5.09%) due to the removal of two hydrated water molecules. The breakdown process involved the elimination of coordinated water molecules at 210 °C, causing a weight loss of 1.98% (Calculated: 2.05%). The removal of four acetate groups generated an endothermic peak at 370 °C with a weight loss of 24.29% (Calculated: 24.38%). The complex exhibited an endothermic peak at 580 °C due to its melting point. Finally, exothermic peaks were observed at 410, 465, 650, and 830 °C, indicating a slow oxidative thermal breakdown, resulting in a weight loss of 22.88% (Calculated: 23.33%) and yielding 2MnO^37^. The thermogram of the Mn(II)/Zn(II) combination (9) indicated a disintegration process occurring in six phases. The first phase occurred at 48°C, with no weight loss observed, but accompanied by an endothermic peak, potentially attributed to the breaking of hydrogen bonds. The second phase occurred at 85 °C, resulting in a weight loss of 3.36% (Calculated: 3.42%) and an endothermic peak, which could be related to the removal of two hydrated water molecules. The breakdown stage, caused by the removal of coordinated water molecules, occurred at 140 °C, resulting in a weight loss of 3.50% (Calculated: 3.55%)^37^. The removal of four acetate groups could explain the endothermic peak reported at 280 with 24.02% weight loss (Calc. 24.11%). Because of its melting point, the complex displayed an endothermic peak at 380°C. Finally, exothermic peaks arise at 415, 550, 680, and 745 °C, corresponding to oxidative thermal breakdown that progresses slowly, leaving with 20.38% weight loss (Calc 20.47%) of MnO and ZnO^38,39^. According to the thermogram of Cu(II) complex (11) the complexes disintegrated in six phases. The first occurred at 48 °C with no weight loss as an endothermic peak, which could be attributed to hydrogen bonds breaking. The second phase occurred at 85 °C, with a weight loss of 3.38% (Calc. 3.74%) as an endothermic peak, which might be attributed to the removal of two hydrated water molecules. The elimination of coordinated water molecules happened during the breakdown process, which occurred at 190 °C with 3.81% weight loss (Calc. 3.88%). The removal of four acetate groups resulted in an endothermic peak at 315C^o^ with 15.85% weight loss (Calc. 15.92%). Because of its melting point, the complex displayed an endothermic peak at 380 °C. Finally, exothermic peaks developed at 439, 460, and 580 °C, corresponding to gradual oxidative thermal breakdown, leaving 2CuO with a weight loss of 21.15% (Calc. 21.21%)^40–42^. The thermal data are present in Table 4.

### Magnetic moments

Table 5 displays the magnetic moments of the metal complexes (2)–(11) at room temperature. Copper(II) complexes (2), (8), (10) and (11) have values in the 1.69–1.71 B.M. range, indicating the existence of one unpaired electron in an octahedral structure^42,43^. The nickel(II) complex (3) revealed values in the 3.12 B.M range, indicating an octahedral nickel(II) complex^44^. The low complex values were caused by spin–spin interactions between metal(II) ions^45^. The diamagnetic properties of Zn(II) complexes (5), (8), and (9), Ag(I) complex (7), and Hg(II) complex (4) were observed^44^ Mn(II) complexes (6) and (9) had BM values of 5.61 and 4.92, respectively, confirming octahedral geometry around Mn(II) ions^46^.

### Electronic spectra

Table 5 summarizes the electronic spectrum data for the ligand (1) and its metal complexes in DMF solution. Ligand (1) in DMF solution showed two bands at 280 nm (log€ = 3. 8 × 10^–3^ mol^−1^ cm^−1^) and 315 nm (log€ = 4.25 × 10^–3^ mol^−1^ cm^−1^)^46^. Copper(II) complexes (2), (8), (10) and (11) showed bands in the 265–267 and 280–312 nm ranges, these bands were due to intraligand transitions, however, the bands appeared in the 462–478, 570–578 and 602–610 nm ranges, were assigned to O → Cu, charge transfer, 2B_1_ → 2E and 2B_1_ → 2B_2_ transitions, indicating a distorted octahedral structure^47,48^. Zinc(II) complexes (5), (8), and (9), Silver (I) complex (7), and Hg(II) complex (4) showed bands were due to intraligand transitions. However, nickel(II) complex (3) showed bands at 268–285, 310–395, 475, 590,610 and 776 nm, the first three bands were within the ligand and the other bands are attributable to O → Ni charge transfer, 3A_2_g(F) → 3T_1_g(P)(ν3), 3A_2_g(F) → 3T_1_g(F)(ν2) and 3A_2_g(F) → 3T_2_g(F)(ν1) transitions respectively, indicating an octahedral Ni(II) geometry^49,50^. The ν_2_/ν_1_ ratio was 1.24, which was less than the usual range of 1.5–1.75, indicating a distorted octahedral Ni(II) complex^51,52^. Mn(II) complexes (6) and (9) showed bands at 265, 280, 307, 462, 586, and 612 for complex (6) which complex (9) showed pands at 260, 283, 318, 420, 560, and 605 nm the first bands were intra-ligand transitions, however the other bands corresponded to A_1_g → E_g_, A_1_g → T_2g_, and Ag → T_2g_ transitions which were compatible to Mn(II) octahedral structure^44,52^.

### Electron spin resonance (ESR)

The spectral data for complexes (2), (6), (8), (9), (10), and (11) were provided in Table 6. The spectra of the copper(II) complexes (2), (10), and (11) exhibited the characteristic of a d^9^ configuration, with a ground state of d(x^2^ − y^2^), which is commonly observed in copper (II) complexes.The complexes displayed g|| > g┴ > 2.0023, indicating an octahedral geometry around the copper(II) ion^50,51^. The g-values can be described by the equation G = (g||− 2)/ (g┴ − 2)^50,52^, where G represents the exchange coupling interaction parameter. If G < 4.0, it signifies the presence of a significant exchange coupling, while a G value > 4.0 suggests that the local tetragonal axes are aligned parallel or slightly misaligned. Complexes (2), (10), and (11) exhibited values of 3.0 and 2.83, indicating spin-exchange interactions between Cu(II) ions. This phenomenon is further confirmed by the values of the magnetic moments (see Table 5). The g||/A|| value is also considered as a diagnostic term for stereochemistry^53^. The values of g||/A|| were 210 and 175.8, which are expected for distorted octahedral complexes. The g-values of the copper(II) complexes with a 2B1g ground state (g|| > g┴) can be expressed as K112^54^.

1 $$ {\text{g}}_{||} = { 2}.00{2 }{-} \, ({\text{8K}}^{{2}}  {||} \lambda^\circ /\Delta {\text{Exy}}) $$

2 $$ {\text{g}}_{ \bot } = { 2}.00{2 }{-} \, ({\text{2K}}_{2 } \bot \lambda^\circ /\Delta {\text{Exz}}) $$

where k|| and k_┴_ are the parallel and perpendicular components respectively of the orbital reduction factor (K), λ° is the spin–orbit coupling constant for the free copper, ΔExy and ΔExz are the electron transition energies of 2B_1_g → 2B_2_g and 2B_1_g → 2Eg. From the above relations, the orbital reduction factors (K||, K_┴_, K), which are measure terms for covalency^55,56^, can be calculated. For an ionic environment, K = 1; while for a covalent environment, K < 1. The lower the value of K, the greater is the covalency.

3 $$ {\text{K}}_{ \bot }^{{2}} = \, \left( {{\text{g}}_{ \bot } - { 2}.00{2}} \right) \, \Delta {\text{Exz }}/{2}\lambda {\text{o}} $$

4 $$ {\text{K}}^{{2}} {||} {\text{ = (g}} {||} \, - { 2}.00{2}) \, \Delta {\text{Exy }}/{8}\lambda {\text{o}} $$

5 $$ {\text{K}}^{{2}} = \, \left( {{\text{K}}^{{2}} {||} \, + {\text{2K}}_{ \bot }^{{2}} } \right)/{3} $$

K values (Table 6), for the copper(II) complexes (2) and (10) were indicating for a covalent bond character^57–60^. Kivelson and Neiman noted that, for ionic environment g|| ≥ 2.3 and for a covalent environment g||< 2.357. Theoretical work by Smith^18^ seems to confirm this view. The g-values reported here (Table 6) showed considerable covalent bond character^59^. Also, the in-plane σ-covalency parameter, α2(Cu) was calculated by

6 $$ \alpha^{{2}} \left( {{\text{Cu}}} \right) \, = \, \left( {{\text{A}}||/0.0{36}} \right) \, + \left( {{\text{g}}|| - {2}.00{2}} \right) \, + {3}/{7}\left( {{\text{g}}_{ \bot } - {2}.00{2}} \right) \, + 0.0{4} $$

The calculated values (Table 6) suggested a covalent bonding^55,56,58–60^. The in-plane and out of- plane π- bonding coefficients β^2^_1_ and β^2^ respectively, are dependent upon the values of ΔExy and ΔExz in the following equations^45^:

7 $$ \alpha^{{2}} \beta^{{2}} = \, \left( {{\text{g}}_{ \bot } - { 2}.00{2}} \right) \, \Delta {\text{Exy}}/{2}\lambda {\text{o}} $$

8 $$ \alpha^{{2}} \beta_{{1}}^{{2}} = \, \left( {{\text{g}}|| \, - { 2}.00{2}} \right) \, \Delta {\text{Exz}}/{8}\lambda {\text{o}} $$

In this work, the complexes (2), and (10) showed β_1_^2^ values 0.82, and 0.69 indicating a covalency in the in-plane π-bonding^54,58–60^. β^2^ value for complexes (2), and (11) showed 0.83 and 0.92 indicating ionict character of the out-of-plane^35^. It is possible to calculate approximate orbital populations ford orbitals^40^ by

9 $$ {\text{A}}|| \, = {\text{ A}}_{{{\text{iso}}}} {-}{\text{ 2B}}\left[ {{1 } \pm \, \left( {{7}/{4}} \right) \, \Delta {\text{g}}||} \right] \, \Delta {\text{g}}_{||} = {\text{ g}}_{||} - {\text{ ge}} $$

10 $$ {\text{a}}^{{2}} {\text{d }} = {\text{2B}}/{\text{ 2B}}^\circ $$

The calculated dipolar coupling for unit occupancy of the d orbital is denoted as A° and 2B°, respectively. During the data analysis, all sign combinations of the Cu hyperfine coupling components were taken into account. Physically meaningful results are only obtained when A|| and A^ are negative. The resulting isotropic coupling constant is also negative, as well as the parallel component of the dipolar coupling 2B (− 175.7 and − 177.2 G). These outcomes can only be observed for an orbital involving the (dx^2^ − y^2^) atomic orbital on copper. The value for 2B is within the normal range for copper(II) complexes. The |Aiso| value is relatively small. By dividing the 2B value by 2B° (the calculated dipolar coupling for unit occupancy of d(x^2^ − y^2^) (235.11 G), using Eq. (10), it is suggested that all orbital populations are 74.75% and 94.41% d-orbital spin density, indicating the presence of the dx^2^ − y^2^ orbital for the unpaired electron^47^. On the other hand, the Co(II) complex (4), Mn(II) complexes (6) and (9), and [Cu(II)/Zn (II)] complex (8), as well as the Cu(II) complex (10), exhibit isotropic values of 2.02, 2.03, and 2.13, respectively, indicating an octahedral structure with covalent bond character^35^^**.**^

### Transmission electron microscopic characterization (TEM)

**Figure 4:**
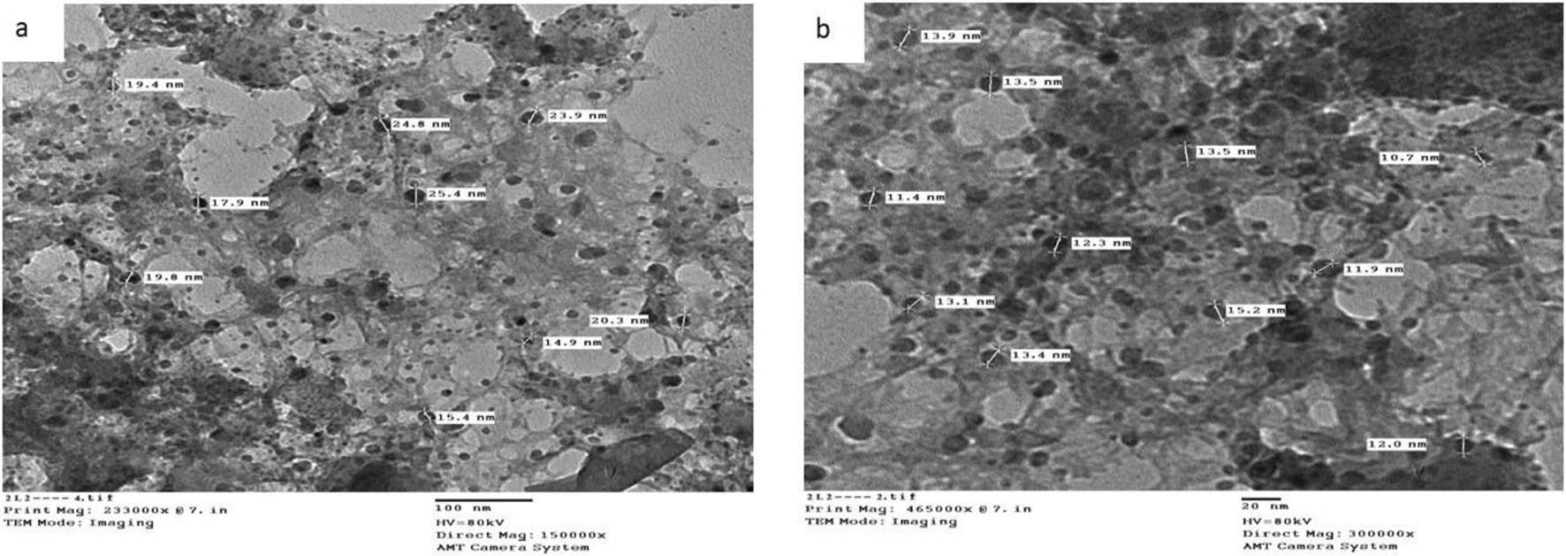
(**a**, **b**) TEM image for Cu(II) (complex(2)) nanoparticles.

**Figure 5:**
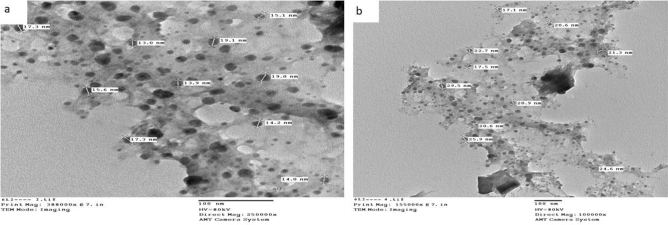
(**a**, **b**) TEM image for Ag(I) (complex (7)) nanoparticles.

**Figure 6:**
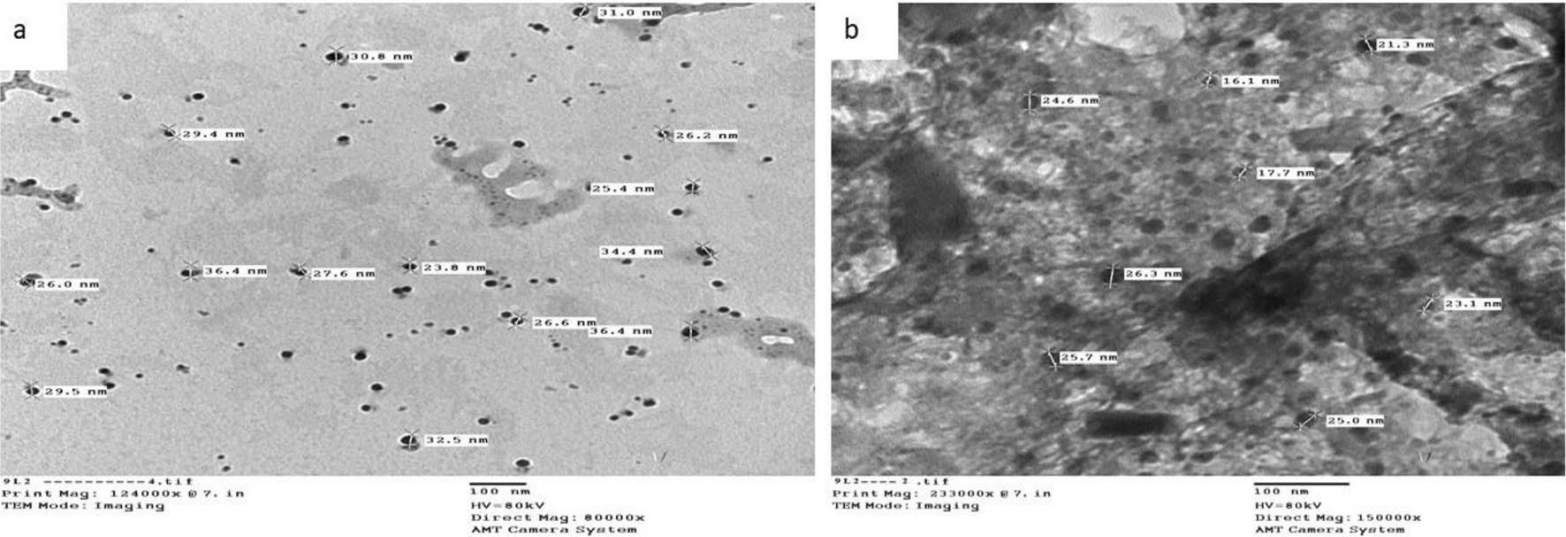
(**a**, **b**) TEM image for Cu(II)/Zn (complex(8)) nanoparticles.

The average diameter of the complexes particles under investigation was determined to be in the 13.0–36.4 nm range, as shown in Figs. 4, 5, and 6. The particles range in size from 1 to 100 nm. The complexes exhibited improved size dependent characteristics. Increased active agent surface area results in faster dissolution of the active agent in an aqueous environment such as the human body, faster dissolution, with gore^61^. The TEM images for Cu(II) complex (2), Ag(I) complex (7), and Cu(II)/Zn complex (8) were showed in Figs. 4, 5 and 6 respectively.

**Figure 7:**
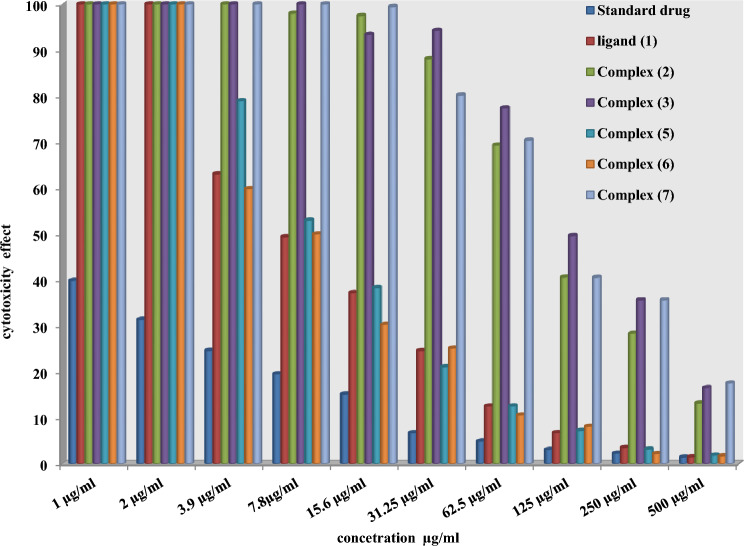
Evaluation of cytotoxicity of metal complexes against human HepG-2 cell line.

**Figure 8:**
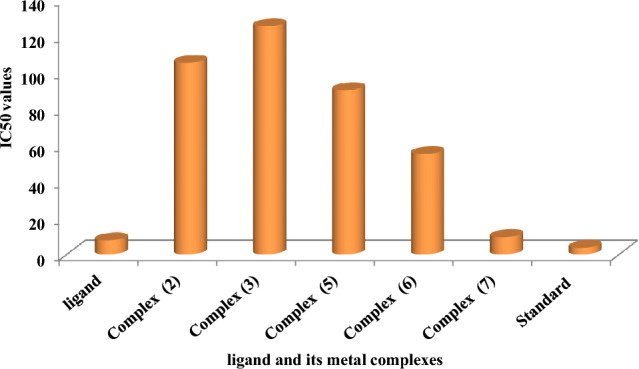
IC50 values of the ligand, and some of its metal complexes against human HEPG-2 cell lines.

Chemotherapeutic investigations: The data presented in Figs. 7, 8 and 13) depict the biological efficacy of ligand (1) and its metal complexes (2), (3), (5), (6), and (7) against HEPG-2 cell lines. In this study, we aim to compare the chemotherapeutic potential of the evaluated complexes with the reference medication as shown in Fig. 7, Cisplatin. Altering the anion, coordination sites, and metal ion type seems to impact the biological behavior by affecting the ability to bind DNA^51,52,62^. Gaetke and Chow previously proposed that metals may induce oxidative tissue damage through a free-radical mediated pathway similar to the Fenton reaction. It has been demonstrated that Cu(I) complexes are capable of forming GSSG (Glutathione disulfide) through the following reactions^47–49^:

11 $$ \left[ {{\text{Cu}}\left( {\text{I}} \right) \, \left( {{\text{ligand}}} \right)} \right] \, + {\text{ GSH }} \to \, \left[ {{\text{Cu}}\left( {\text{I}} \right) \, \left( {{\text{GS}}} \right)} \right] \, + {\text{ ligand }} + {\text{H}}^{ + } $$

12 $$ \left[ {{\text{Cu}}\left( {\text{I}} \right)\left( {{\text{GS}}} \right)} \right] \, + {\text{GS}} + \, [{\text{Cu }}\left( {{\text{II}}} \right) + {\text{GSSG]}} $$

The DNA-bound Cu(II) complex undergoes oxidation in the presence of H_2_O_2_, potentially generating Cu(II) (oxo/hydroxo) species^54–57^. As a consequence, the interaction between the Cu(II) complex and nucleic acid occurs through the Cu-oxo/hydroxy intermediate. Cu(II) complexes are well-known for their redox activity, which seems to be involved in biological processes^58–60^. The conversion of Cu(II) to Cu(I) by intracellular thiols, such as GSH (glutathione, a nonenzymatic antioxidant in environments containing oxygen), forms the basis for the redox cycling of Cu complexes^61^. Equations (13–15) illustrate the chemical pathway for [Cu(II) (ligand)] complexes. To summarize, Cu (II) complexes readily combine with GSH^42^, resulting in the formation of Cu(I) complexes and GS+. This Cu(I) complex can generate a superoxide anion in the presence of oxygen, which can induce ROS through a fenton-like reaction:

13 $$ \left[ {{\text{Cu }}\left( {{\text{II}}} \right) \, \left( {{\text{ligand}}} \right)} \right] \, + {\text{ GSH }} \to \, \left[ {\left( {{\text{GS}}} \right){\text{ Cu}}\left( {{\text{II}}} \right) \, \left( {{\text{ligand}}} \right)} \right] \, + {\text{ H}}^{ + } $$

14 $$ \left[ {\left( {{\text{GS}}} \right){\text{ Cu}}\left( {{\text{II}}} \right) \, \left( {{\text{ligand}}} \right)} \right] \, \to \, \left[ {{\text{GS}}* \, + \, } \right[{\text{Cu}}\left( {\text{I}} \right) \, \left( {{\text{ligand}}} \right)] $$

15 $$ \left[ {{\text{Cu}}\left( {\text{I}} \right) \, \left( {{\text{ligand}}} \right)} \right] \, + {\text{ O}}_{{2}} \to {\text{ O}}_{{2}} \, + \, \left[ {{\text{Cu}}\left( {{\text{II}}} \right) \, \left( {{\text{ligand}}} \right)} \right] $$

As previously described^51^, treatment with the different complexes in DMSO had an equivalent impact on the utilized tumoral cell line. The solvent dimethyl sulphoxide (DMSO) did not influence cell growth. The ligand (1) exhibited a mild inhibitory effect at the tested concentrations, while the complexes had a more significant effect on HEPG-2 cell lines. The ratio of surviving fraction against MCF-7 tumor cells increased as the concentration decreased within the examined concentration range^50^. The cytotoxicity data indicated that the complex (7) under investigation were effective. complex (7) was the most detrimental to cell lines, with a half-maximal inhibitory concentration (IC50) value of 9.5 M, followed by complex (6) with an IC50 value of 55 Complex (7) exhibited cytotoxicity against cell lines at all doses compared to the standard medication (Cisplatin), which had an IC50 value of 15.3 M. This can be attributed to the Cu(II) ion being bound to DNA. Since altering the anion and type of metal ion can impact biological behavior due to differences in DNA binding ability, the evaluation of different complexes is particularly intriguing from this perspective. Tweedy's chelation theory suggests that the chemotherapeutic activity of the complexes can be traced back to the central metal atom^48^. Additionally, the positive charge of the metal raises the acidity of the coordinated ligand containing protons, resulting in stronger hydrogen bonds that enhance biological activity^39^. The cytotoxic effect of the ligand and some of its metal complexes on HEPG-2 cell lines is presented in Table 7, Fig. 8.

**Table 7:** Cytotoxic effect of the ligand and some of its complexes against HEPG-2 cell line.

Concentration (µg/ml)	Order of cytotoxic effect of studied complex
	(HEPG-2 cell line)
500	(7) > (3) > (2) > (5) > (6) std
250	(7) = (3) > (2) > (1) > (2) > (5) > std
62.5	(3) > (7) > (2) > (5) = (1) > (2) > std
15.6	(7) > (2) > (3) > (5) > (2) > (6) std
3.9	(7) > (2) > (3) > (5) > (1) > std

**Figure 9:**
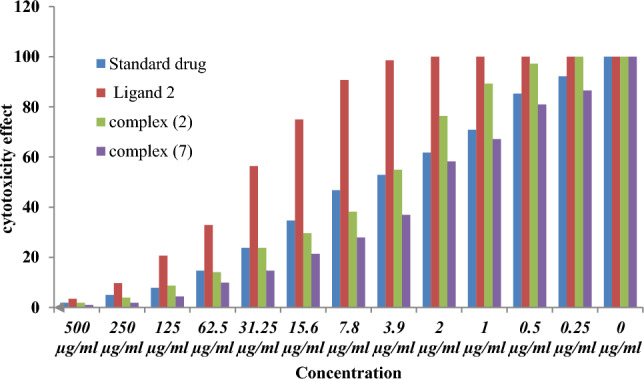
Evaluation of cytotoxicity of metal complexes against human MCF-7 cell line.

**Figure 10:**
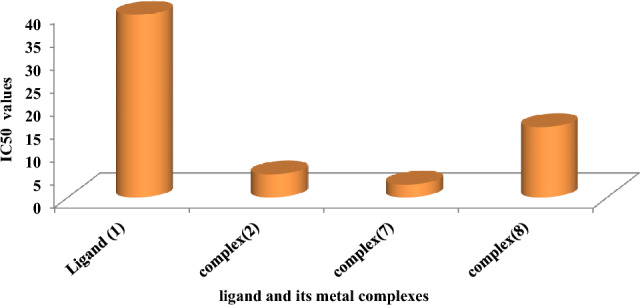
IC50 values of the ligand, and some of its metal complexes against human MCF-7 cell lines.

**Figure 11:**
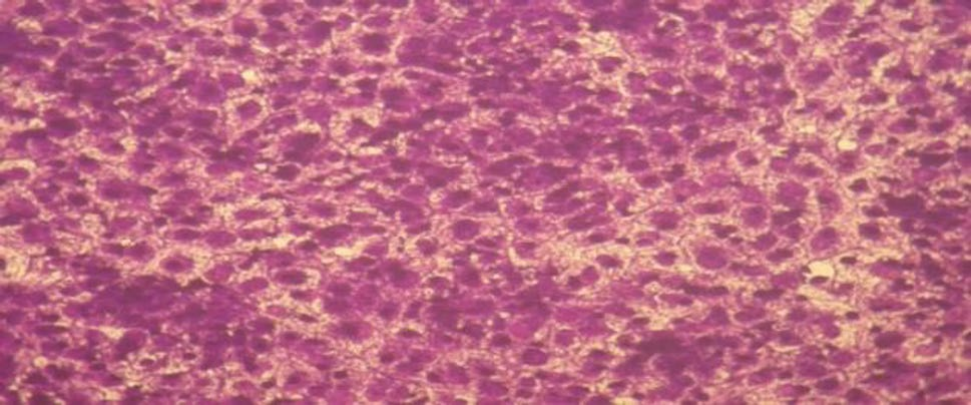
MCF-7 cell lines of non-treated (control).

**Figure 12:**
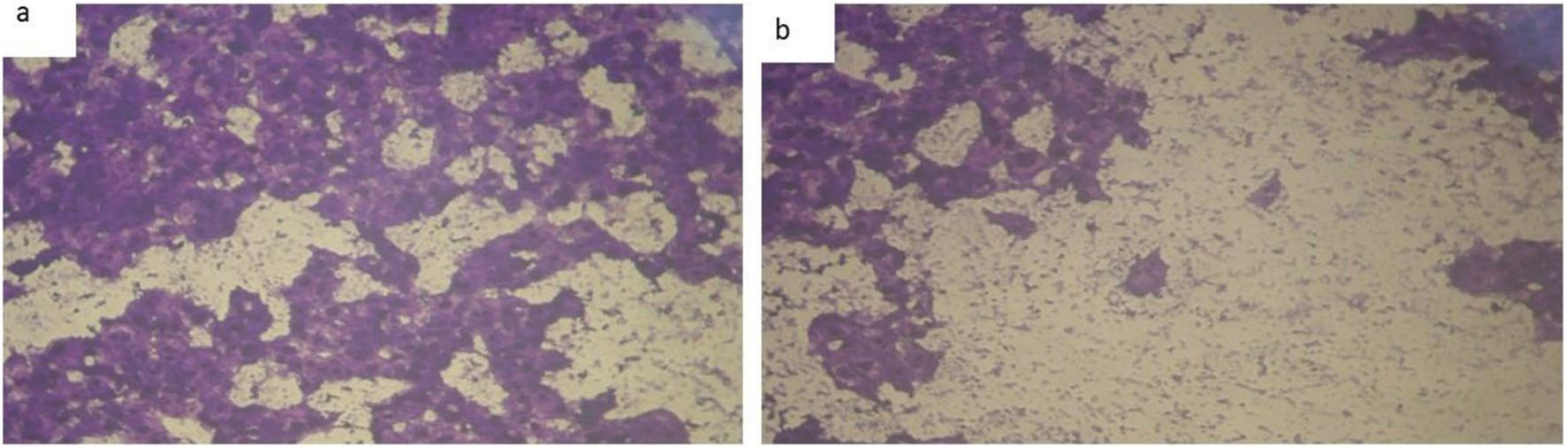
(**a**) MCF-7 cell lines of treated with complex (2) at 10 µg/ml concentration and (**b**) MCF-7 cell lines of treated with complex (2) at 100 µg/ml concentration.

Chemotherapeutic investigations: The data presented in Figs. 9, 10, 11, 12 depict the biological efficiency of ligand (1) and its metal complexes (2), (7), and (8) against MCF-7 cell lines. In this study, we aim to compare the chemotherapeutic potential of the evaluated complexes with the reference medication, Cisplatin. Altering the anion, coordination sites, and metal ion type seems to impact the biological behavior by affecting the ability to bind DNA^51,52,62^. Gaetke and Chow previously proposed that metals may induce oxidative tissue damage through a free-radical mediated pathway similar to the Fenton reaction.

**Figure 13:**
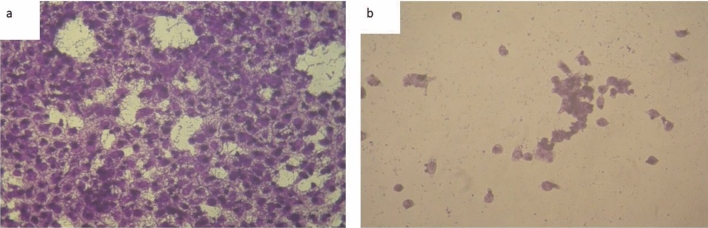
(**a**) HEPG-2 cell lines of treated with complex (7) at 1 µg/ml concentration and (**b**) HEPG-2 cell lines of treated with complex (7) at 100 µg/ml concentration.

The DNA-bound Cu(II) complex undergoes oxidation in the presence of H_2_O_2_, potentially generating Cu(II) (oxo/ hydroxo) species^54–57^. As a consequence, the interaction between the Cu(II) complex and nucleic acid occurs through the Cu-oxo/hydroxy intermediate. Cu(II) complexes are well-known for their redox activity, which seems to be involved in biological processes^58–60^. The conversion of Cu(II) to Cu(I) by intracellular thiols, such as GSH (glutathione, a nonenzymatic antioxidant in environments containing oxygen), forms the basis for the redox cycling of Cu complexes^61^, as shown before. Treatment with the different complexes in DMSO had an equivalent impact on the utilized tumoral cell line. The solvent dimethyl sulphoxide (DMSO) did not influence cell growth. The ligand (2) exhibited a mild inhibitory effect at the tested concentrations, while the complexes had a more significant effect on MCF-7 cell lines. The ratio of surviving fraction against MCF-7 tumor cells increased as the concentration decreased within the examined concentration range^50^. The cytotoxicity data indicated that the complexes (2) and (8) under investigation were effective, copper(II) complex (2) was the most detrimental to cell lines, with an IC50 value of 5.0 M, followed by complex (7) with an IC50 value of 2.8, complex (7) exhibited cytotoxicity against cell lines at all doses compared to the standard medication (Cisplatin), Fig. 13, which had an IC50 value of 15.3 M. The IC50 value is 15.3 M Complex (8), Figs. 8 and 9 also displayed cytotoxicity against cell lines. This can be attributed to the Cu(II) ion being bound to DNA. Since altering the anion and type of metal ion can impact biological behavior due to differences in DNA binding ability, the evaluation of different complexes is particularly intriguing from this perspective. Tweedy's chelation theory suggests that the chemotherapeutic activity of the complexes can be traced back to the central metal atom^48^. Additionally, the positive charge of the metal raises the acidity of the coordinated ligand containing protons, resulting in stronger hydrogen bonds that enhance biological activity^39^. The cytotoxic effect of the ligand and some of its metal complexes on MCF-7 cell lines is presented in Table 8, Figs. 9 and 10.

**Table 8:** Cytotoxic effect of the ligand and some of its complexes against MCF-7 cell line.

Concentration (µg/ml)	Order of cytotoxic effect of studied complex
	(MCF-7 cell line)
500	(7) > std = (2) > ligand > (8)
250	(7) > (2) > std > ligand > (8)
62.5	(7) > (2) > std > (8) > ligand
15.6	(7) > (2) > std > ligand > (8)
3.9	(7) > std > (2) > (8) > ligand
1.0	(7) > std > (2) > (8) > ligand

### Molecular docking of some complexes with breast cancer protein 3S7S

Molecular modeling, docking is a method which predicts the preferred orientation of one molecule to a second when a ligand and a target are bound to each other to form a stable complex. Knowledge of the preferred orientation in turn may be used to predict the strength of association or binding affinity between two molecules using, for example, scoring functions.The protein 3S7S, which has been selected, serves as a representation of the crystal structure of the chemical that is considered to be physiologically active in the human placenta. Through the utilization of this approach, one is able to ascertain the site at which the ligand-receptor interaction occurs, as well as the type of interaction that takes place. Furthermore, this method also enables the calculation of the distance between the ligand and the receptor within the interaction grid. The scoring energy obtained from the docking computations corresponds to the inhibitory effect exhibited by the relevant ligand. Within the context of the present investigation, the protein 3S7S mirrors the crystal structure of the human placental aromatase enzyme, which is responsible for the synthesis of the estrogen hormone and contributes to estrogen-dependent breast cancer50. Based on the scoring energy, it can be observed that all ligands possess a considerable number of interactions with the receptor protein. The outcomes of the study serve to demonstrate the capacity of the ligand to impede the protein 3S7S. Based on the scoring energy, it can be observed that all ligands possess a considerable number of interactions with the receptor protein. The outcomes of the study serve to demonstrate the capacity of the ligand to impede the protein 3S7S.

#### Complex 2

**Figure 14:**
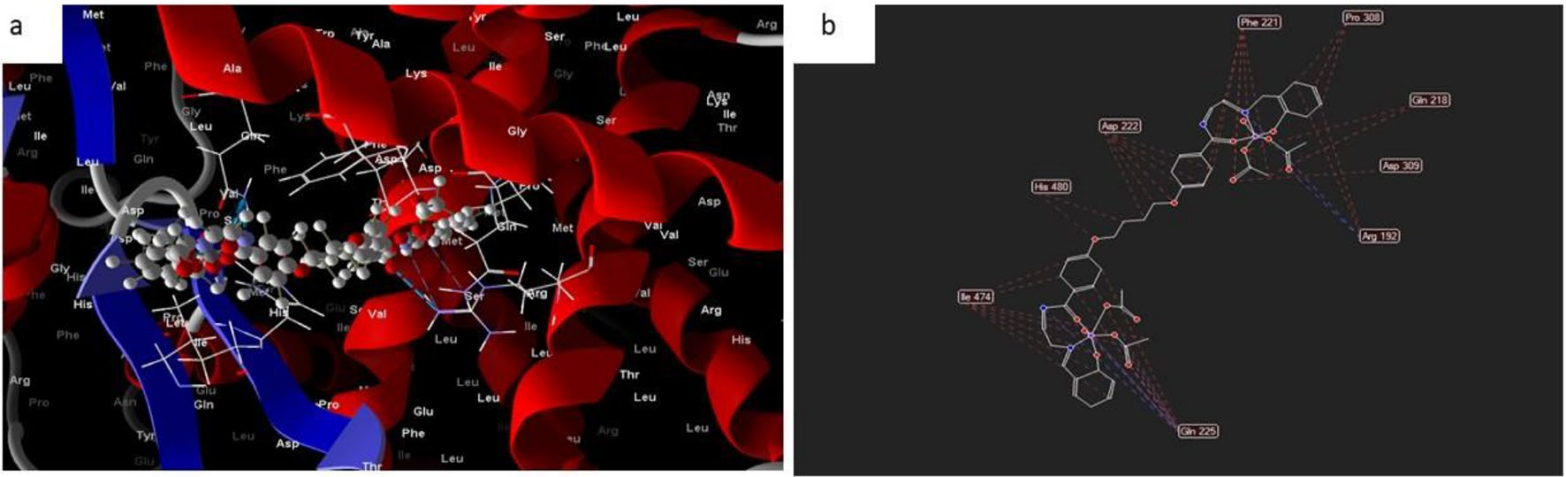
(**a**) Virtual molecular docking of the best docked complex (2) with 3S7S protein and (**b**) 2D structure of molecular docking of complex(2) with 3S7S protein.

In the case of the docked complex 2, it can generally be observed that the distances associated with the effective ligand-receptor interactions, as depicted docked and 2D structure in Fig. 14 in complex (2) are approximately 3.5 A. This indicates the presence of typical actual bonds, thus signifying a significant binding affinity. The nearest interaction, for instance, is identified through H-donors with 3S7S, with a distance of 2.18 A, while complex2 exhibits a scoring energy (S) of 182,278 kcal. Additionally, it was determined that complex2 possesses 17 binding sites comprising various amino acids (Gln 225, lIe 474, His 480, Asp 222, Phe 221, Pro 308, Gln 218, Asp 309, and Arg 192), suggesting their considerable inhibitory effect.

#### Complex (3)

**Figure 15:**
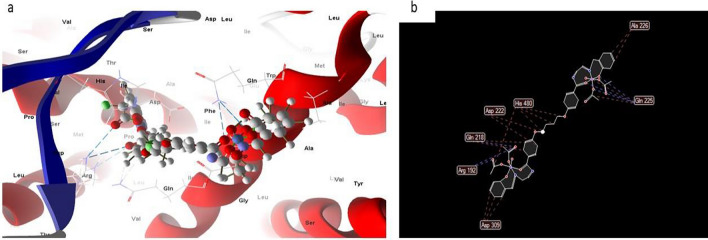
(**a**) Virtual molecular docking of the best docked complex (3) with 3S7S protein and (**b**) 2D structure of molecular docking of complex (3) with 3S7S protein.

While most docked and 2D structure of complex (3), Fig. 15 had effective ligand-receptor interaction lengths of 3.5 A, indicating the presence of typical real bonds and thus strong binding affinity. The nearest interaction, for example, is observed via H-donors with 3S7S (2.34 A) and (complex (3) With scoring energy (S) 195,127 kcal. Furthermore, seventeen binding sites of distinct amino acids (Ala 226,Gln 225, Asp 309, Arg 192, Gln 218, Asp 222, and His 480) with complex(3) were found, suggesting their higher inhibition than copper complex (2).

#### Complex 5

**Figure 16:**
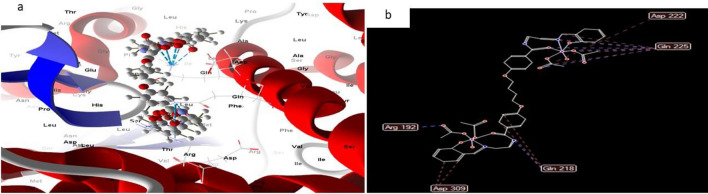
(**a**) Virtual molecular docking of the best docked complex (5) with 3S7S protein and (**b**) 2D structure of molecular docking of complex (5) with 3S7S protein.

However, in most cases, the docked and 2D structure of complex (5), Fig. 16 have effective ligand-receptor interaction distances of 3.5 A, indicating the presence of typical actual bonds and thus strong binding affinity. The nearest interaction, for example, is observed via H-donors with 3S7S (2.46 A) and complex (5) With scoring energy (S) 233,009 kcal. Furthermore, seventeen binding sites for various amino acids (Gln 218, Asp 309, Arg 192, Asp 222, and Gln 225) with complex (5) were discovered.

#### Complex 6

**Figure 17:**
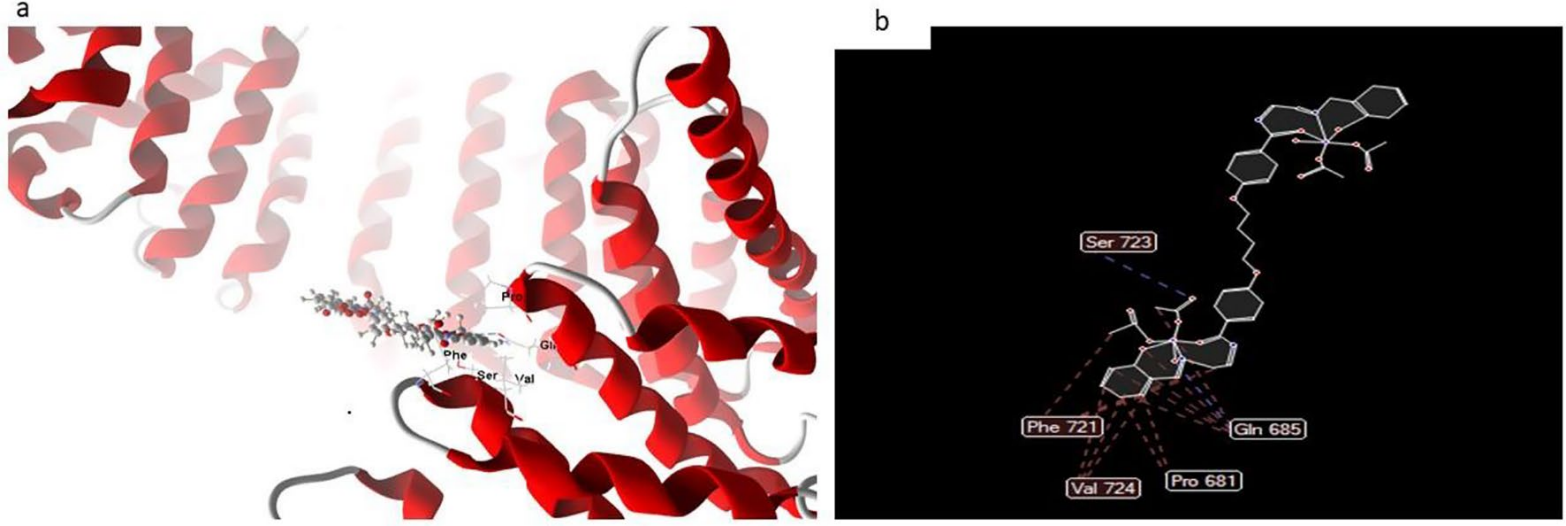
(**a**) Virtual Molecular docking of the best docked complex (6) with 3S7S protein and (**b**) 2D structure of molecular docking of complex(6) with 3S7S protein.

In most cases, the docked and 2D structure of complex (6) in Fig. 17, ligand-receptor interaction distances were 3.5 A, showing the presence of typical real bonds and consequently considerable binding affinity. For example, the nearest contact is found using H-donors with 3S7S (3.28 A) and complex (6) with scoring energy (S) 230,220 kcal. Furthermore, with compound (6), which is regarded to be the most effective complex towards 3S7S protein, seventeen binding sites of different amino acids (Gln 685, Pro 681, Val 724, Phe 721, and Ser 723) were found.

#### Complex 7

**Figure 18:**
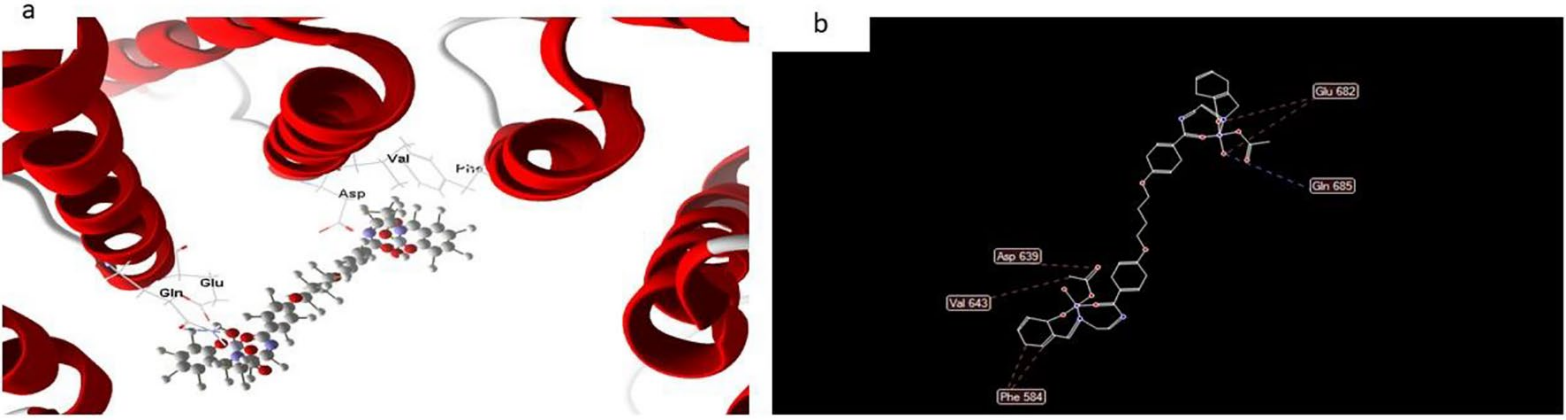
(**a**) Virtual Molecular docking of the best docked complex (7) with 3S7S protein and (**b**) 2D structure of molecular docking of complex (7) with 3S7S protein.

However, the docked and 2D structure of complex (7) in Fig. 18 have effective ligand-receptor interaction distances were ≤ 3.57 A in most cases, which indicates the presence of typical real bonds and hence high binding affinity. For example, the nearest interaction is observed via H-donors with 3575 (3.35 A) and complex (7) with scoring energy (S) 173,468 kcal Furthermore, seventeen binding sites were observed of different amino acids (Gln 685, Glu 682, Phe 584, Val 643 and Asp 639) with complex (7).

Hence we can conclude that the inhibition of complex5 > complex 6 > complex 3 > complex 2 > complex 7 to breast cancer protein 3S7S.

### Molecular docking of some complexes with liver cancer protein 4OO6

4OO6 represents Crystal structure of human KAP-beta2 bound to the NLS of HCC1 (Hepato Cellular Carcinoma protein 1) This approach elucidates the ligand-receptor site and type of interactions. It also gives an estimation of the distance between the ligand and the receptor inside the interaction grid. The scoring energy of each pose simulated by the docking calculations reflects the degree of inhibition effect of the corresponding ligand. In the present study.

#### Complex 8

**Figure 19:**
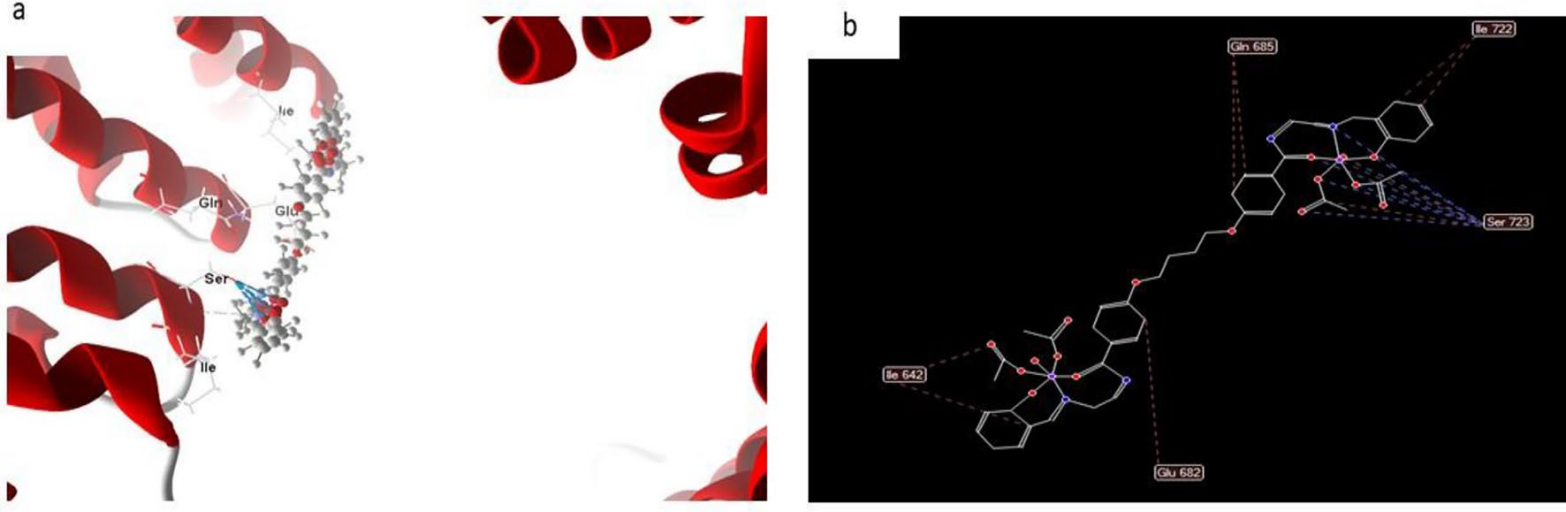
(**a**) Virtual Molecular docking of the best docked complex (8) with 4OO6 protein and (**b**) 2D structure of molecular docking of complex (8) with 4OO6 proteins.

In most cases, the docked and 2D structure of complex (8), Fig. 19 had effective ligand-receptor interaction distances of 3.1 A, indicating the presence of typical actual bonds and thus strong binding affinity. The nearest interaction, for example, is observed via H-donors with 4OO6 (2.17 A) and (complex (8) With scoring energy (S) 244,215 kcal. Furthermore, twelve binding sites of distinct amino acids (Gln 685, lle 722, lle 642, Glu 682, and Ser 723) with complex (8) were found, suggesting their strong inhibition.

#### Complex 9

**Figure 20:**
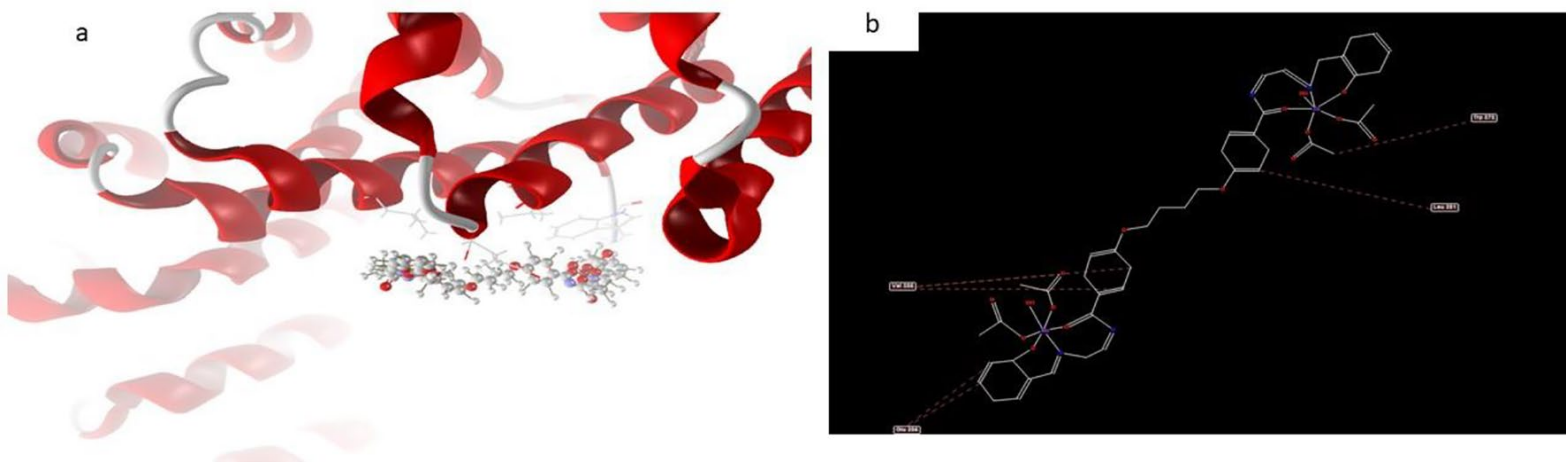
(**a**) Virtual molecular docking of the best docked complex (9) with 4OO6 protein and (**b**) 2D structure of molecular docking of complex (9) with 4OO6 protein.

However, the docked and 2D structure of complex (9), Fig. 20 have effective ligand-receptor interaction distances were ≤ 3.1 A in most cases, which indicates the presence of typical real bonds and hence high binding affinity. For example, the nearest interaction is observed via H-donors with 4OO6 (2.17 A) and complex (9) with scoring energy (S) 229,215 kcal Furthermore, 17 binding sites were observed of different amino acids (Glu 286, Val 386, lle 642, Leu 281 and Trp 373) with complex (9) demonstrating their high inhibition.

#### Complex 10

**Figure 21:**
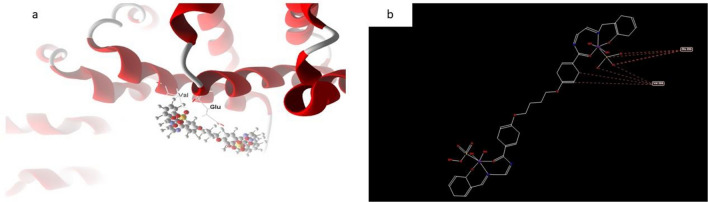
(**a**) Virtual Molecular docking of the best docked complex (10) with 4OO6 protein and (**b**) 2D structure of molecular docking of complex (10) with 4OO6 protein.

In contrast, the docked and 2D structure of complex (10), Fig. 21 has effective ligand-receptor interaction lengths of 4.15 A in most cases, indicating the presence of typical actual bonds and thus strong binding affinity. The nearest interaction, for example, is detected via H-donors with 4OO6 (3.85A) and (complex10), with scoring energy (S) 195,131 kcal. Furthermore, twelve binding sites of various amino acids (Val 336 and Glu 286) were discovered, with complex10 exhibiting their reduced inhibition^52–55,57,58,61,62^.

Hence we can conclude that the inhibition of complex8 > complex 9 > complex 10 to Hepato Cellular Carcinoma protein.

## Conclusion

New metal complexes were created in this investigation. The ligand adopted a hex dentate ligand design, while the metal complexes adopted a tetragonal distorted octahedral geometry around metal ions, according to structural and spectroscopic features. Molar conductance studies indicate that all of the complexes are non-electrolytic in nature. The ligand is coupled to the center metal ion, resulting in six-membered rings containing metal ions. The anticancer actions of the ligand as well as some of its metal complexes were evaluated. The toxicity of both the ligand and the metal complexes was shown to be concentration dependent, with increasing complex concentration decreasing cell viability.

## Data Availability

The datasets used and/or analyzed during the current study available from the corresponding author on reasonable request.
